# Lambs Weaned Early onto a Herb-Clover Mix Have the Potential to Grow at a Similar Rate to Unweaned Lambs on a Grass-Predominant Pasture

**DOI:** 10.3390/ani10040613

**Published:** 2020-04-02

**Authors:** Lukshman Jay. Ekanayake, Rene Anne Corner-Thomas, Lydia Margaret Cranston, Paul Richard Kenyon, Stephen Todd Morris

**Affiliations:** School of Agriculture and Environment, Private Bag 11-222, Massey University, Palmerston North 4442, New Zealand; R.Corner@massey.ac.nz (R.A.C.-T.); L.Cranston@massey.ac.nz (L.M.C.); P.R.Kenyon@massey.ac.nz (P.R.K.); S.T.Morris@massey.ac.nz (S.T.M.)

**Keywords:** early weaning, lamb growth, plantain, chicory, clover

## Abstract

**Simple Summary:**

Early weaning of lambs may be a useful management tool when either herbage quality or quantity limits lamb growth. Herb-clover mixes containing chicory, plantain, red clover, and white clover have been shown to improve the growth of suckling lambs and those weaned at a traditional age compared to grass-predominant pastures in New Zealand. In this study, lambs were weaned at a minimum live weight of 14 kg and their liveweight gains at a conventional weaning age (~99 days of age) were compared with lambs unweaned on a grass-predominant pasture. We found that lambs weaned early onto a herb-clover mix have the potential to achieve live weights similar to lambs unweaned on grass-predominant pasture.

**Abstract:**

Liveweight gain of lambs weaned early at a minimum live weight of 14 kg, at ~50 days of age, onto a herb-clover mix was compared with lambs that remained unweaned on a grass-predominant pasture or a herb-clover mix until conventional weaning (at ~99 days of age). Over two years, twin sets of lambs that had a minimum live weight of 14 kg were randomly allocated to one of three treatments: (1) Early weaning of lambs onto a herb-clover mix (Herb_EW_); (2) ewes and lambs grazing a herb-clover mix until conventional weaning (Herb_CW_); and (3) ewes and lambs grazing a grass-predominant pasture until conventional weaning (Grass_CW_). Herb_EW_ lambs had slower (*p* < 0.05) growth rates than Grass_CW_ lambs between early weaning and conventional weaning in 2016 and were 800 g lighter (*p* < 0.05) at conventional weaning. In 2017, however, both Herb_EW_ and Grass_CW_ had similar (*p* > 0.05) growth rates and did not differ (*p* > 0.05) in live weight at conventional weaning. Herb_CW_ lambs had a greater (*p* < 0.05) growth rates than both Herb_EW_ and Grass_CW_ lambs in both years. Lambs weaned early onto a herb-clover mix have the potential to achieve live weights similar to lambs unweaned on grass-predominant pasture. Further research, however, is required to understand conditions under which early-weaned lambs can achieve similar live weights.

## 1. Introduction

In New Zealand, herb-clover mixes containing plantain (*Plantago lanceolata*), chicory (*Cichorium intybus*), red clover (*Trifolium pratense*), and white clover (*Trifolium repens*) have been shown to increase the growth rate of both unweaned [1,2,3] and weaned lambs [4,5], and are commonly used by farmers [6]. Greater liveweight gains of lambs between four and eight months of age on the herb-clover mix are driven by greater nutritional quality (greater protein and metabolizable energy content and low fiber content) compared with grass-predominant pastures [4,5,7]. It is also known that white clover, red clover, and chicory have greater feeding values than ryegrass [8]. Further, studies indicate that chicory, red clover and plantain are preferentially grazed by lambs compared to ryegrass [9]. Lambs offered herb-clover mixes, therefore, can have greater voluntary feed intakes compared with lambs offered ryegrass pastures [10].

Early weaning of lambs at approximately 50 days of age is a potential tool that farmers can use to reduce the overall flock feed demand, by allowing ewes to cease lactating and return to a maintenance feeding levels. Lambs weaned early at a minimum live weight of 16 kg, at approximately 60 days of age, onto a herb-clover mix have been shown to achieve similar or improved liveweight gains to a conventional weaning age compared with lambs that remained with their dam on a grass-predominant pasture [11]. It is, however, not known if lambs can be successfully weaned at a younger age (~50 days of age) and at minimum live weight of 14 kg onto a herb-clover mix and still achieve similar growth rates as unweaned lambs grazing on a grass-predominant pasture. The aim of the current study was to compare the liveweight gain of lambs weaned early at approximately 50 days of age, at a minimum live weight of 14 kg, onto a herb-clover mix with lambs who remained unweaned on a herb-clover mix or grass-predominant pasture. It was hypothesized that lambs weaned early onto a herb-clover mix could achieve similar growth rates to unweaned lambs on grass-predominant pasture.

## 2. Materials and Methods

### 2.1. Herbage Treatments

Eight paddocks (13.85 ha in total land area) of herb-clover mix containing plantain, chicory, red clover, white clover, and seven paddocks (15.85 ha in total land area) of grass-predominant pasture (perennial ryegrass (*Lolium perenne* L.) and white clover) were used for the duration of the study in both years (2016 and 2017). During the experimental period (August to December), both herbage types were managed using rotational grazing to provide *ad-libitum* intakes such that post-grazing sward surface heights were maintained at a minimum of five cm in the grass-predominant pasture and seven cm in the herb-clover mix [7,12].

### 2.2. Experimental Design

This study was conducted at Massey University’s Keeble farm, 5 km southeast of Palmerston North, New Zealand (40°24′ S and 175°36′ E) and replicated over two consecutive springs. All manipulations were approved by the Massey University Animal Ethics Committee (MU AEC17/40). Romney ewes that had conceived during a 17-day breeding period and diagnosed as twin bearing using transabdominal ultrasound were selected for the study. Throughout the gestation period, within each year, ewes were managed as a single flock (group) under commercial pastoral farming conditions as part of a larger flock on grass-predominant pastures.

Lambing began on 26th of August in 2016 and 28th of August in 2017. All lambs were weighed, ear tagged, and identified to their dam within 24 h of birth. In each year, lambs and ewes were managed as a single mob on a grass-predominant pasture from the start of lambing (L0) until the start of the study (L53 in 2016 and L51 in 2017). According to veterinary advice, lambs were drenched (1 mL per 5 kg live weight) with an oral triple combination drench (Matrix, Merial Ancare, Manukau City, New Zealand) at L26 in 2016 and L25 in 2017 and thereafter at 28-day intervals throughout the study to control internal parasites.

Twin rearing ewes (n = 75 in 2016 and n = 61 in 2017) with twin sets of lambs of which both had a minimum live weight of 14 kg at L50 in 2016 (range 14.2 to 22.5 kg; n = 150) and at L48 in 2017 (range 14.0 to 24.0 kg; n = 122) were allocated to one of three treatments: (1) Early weaning of lambs at ~50 days of age onto a herb-clover mix (Herb_EW_, n = 54 in 2016 and n = 46 in 2017); (2) ewes and lambs grazing a herb-clover mix until conventional weaning at ~ 99 days of age (Herb_CW_, n = 46 in 2016 and n = 42 lambs in 2017); and (3) ewes and lambs grazing a grass-predominant pasture until conventional weaning (Grass_CW_, n = 50 in 2016 and n = 34 lambs in 2017). Lambs were allocated to treatments using a stratified sampling procedure, based on the live weights of lambs, in order minimize the differences of average lamb live weights between groups.

Lambs and ewes allocated to treatments were managed as a single group on a grass-predominant pasture from the start of lambing (L0) until the start of the study (L53 in 2016 and L51 in 2017). Beginning at L50 in 2016 and L48 in 2017, Herb_EW_ or Herb_CW_ lambs were gradually introduced to herb-clover mix over a four-day period by increasing the duration on the herb-clover mix each day (i.e., 4 h day 1, 8 h day 2, 12 h day 3, and 24 h day 4), prior to the start of the main study. At L50 in 2016 and at L48 in 2017, average live weights of lambs were similar among treatments (17.1 ± 0.8, 17.3 ± 0.6, 16.9 ± 0.4 kg in Herb_EW_, Herb_CW_, and Grass_CW_ in 2016, and 17.9 ± 1.5, 18.4 ± 1.3, 18.6 ± 1.1 kg in Herb_EW_, Herb_CW_, and Grass_CW_ in 2017, respectively). The study began at L53 and L51 in 2016 and 2017, respectively. Once early weaning had been conducted the ewes in the Herb_EW_ treatment were managed with Grass_CW_ ewes and lambs on grass-predominant pasture to L99 in both years. Lambs in the Herb_EW_ treatment were managed with Herb_CW_ ewes and lambs on herb-clover mix to L99 in both years. Ewes develop an exclusive bond with their lambs within 1 to 2 hr of birth and avoid fostering alien lambs for the remainder of the lactation [13]. Therefore, it is unlikely that Herb_CW_ ewes would have allowed Herb_EW_ lambs to suck milk.

### 2.3. Animal Measurements

Lambs and ewes were weighed within 1 h of removal from herbage at L53, L65 and L99 in 2016 and L51, L65 and L99 in 2017. Ewe body condition score (BCS), scale 1–5 including half units [14], was assessed at each weighing by a single operator.

### 2.4. Herbage Measurements

Herbage masses were measured at L47, L65 and L99 in both years of the study. Four random quadrat cuts (0.1 m^2^ each) were taken to ground level from herb-clover mix and grass-predominant pasture at each sampling date using an electric shearing hand-piece [15]. Samples were then oven dried to a constant weight to estimate herbage mass. In addition, four composite herbage samples each containing ten grab samples per herbage type, were also collected at each sampling date to determine the botanical and nutritional composition [15]. To determine the botanical composition of the herbage, a subsample from each composite sample (four per herbage type) was sorted into each species (herb-clover mix: plantain, chicory, red clover, white clover; grass-predominant pasture: ryegrass, clover; other grasses (combined), weeds, and dead matter) and then oven dried and weighed to determine the botanical composition. The remaining sample was then frozen, dried, ground, sieved (1 mm) and analyzed using in vitro methods to determine the nutritional quality. These measures included dry matter digestibility (DMD, [16]), percentage crude protein (CP; “Dumas” procedure, AOAC method 968.06 using a Leco total combustion method, LECO Corporation, St. Joseph, MI, USA). Percentage acid detergent fiber (ADF) was analyzed by a Tecator Fibretec System [17]. Metabolizable energy (ME) content of herbages was calculated from the organic matter digestibility (DOMD × 0.16 MJ/Kg DM, [16]).

### 2.5. Statistical Analysis

Analyses were performed separately for each year due to the differences in the days on which measurements were collected, differences in climate and herbage quality, and animals used between years. The individual animal was considered as the experimental unit for the analyses. Live weight of lambs and ewes were subjected to analysis of variance for repeated measures using the MIXED procedure in SAS (Statistical Analysis System, version 9.2; SAS Institute Inc., Cary, NC, USA). The model used was a two-way interaction of treatment and measurement date to compare treatment groups. The analysis contained and tested contrasts of all treatment and date combinations in order to describe differences among treatments and across time. The model for lamb live weight included the fixed effects of weaning treatment (Herb_EW_, Herb_CW_, Grass_CW_), sex of lamb (male, female), measurement date and the two-way interaction of treatment and measurement date. The live weight of lambs at the start of the treatment period was included in the model as a covariate. The model for lamb liveweight gain included the fixed effects of weaning treatment and sex of lamb. The live weight of lambs at the start of the treatment period was included in the model as a covariate.

The model for ewe live weight included the fixed effects of weaning treatment and measurement date and two-way interaction of treatment and measurement date. The duration from lambing to the start of the treatments was included as a covariate. The model for ewe liveweight gain included the fixed effect of weaning treatment. The live weight of the ewe at the start of the treatment period was included in the model as a covariate. Ewe body condition score was analyzed using a Poisson distribution and logit transformation using the GENMOD procedure in SAS. The Poisson distribution was chosen as it is a nonlinear regression model for discrete outcomes. The model included the fixed effects of weaning treatment and measurement date.

Botanical composition of herbages was analyzed using the MIXED procedure in SAS. The model included fixed effects of plant species, measurement date and year. Herbage masses were analyzed using a model that included herbage type and measurement date as fixed effects. The nutritional quality data were analyzed using the MIXED procedure in a model that included the fixed effects of herbage type and measurement date. In 2016, one lamb died in each of the Herb_EW_ and Herb_CW_ treatments and no lambs died in 2017. In the tables presented, the number of lambs and ewes reported vary between weighing dates due to data recording errors resulting in missing data.

## 3. Results

### 3.1. Botanical Composition, Herbage Mass and Nutritional Quality of Herbage

**Figure 1:**
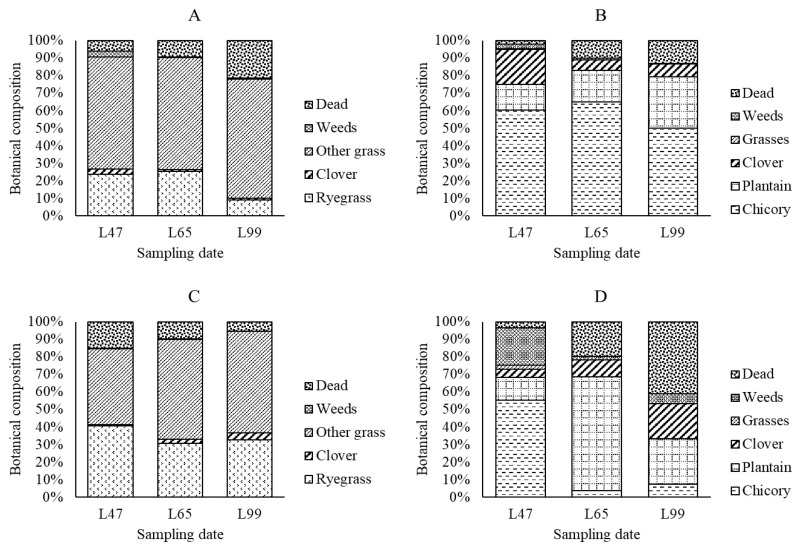
The botanical composition of the components of grass-predominant pasture (**A**), herb-clover mix (**B**) in 2016 and grass-predominant pasture (**C**) and herb-clover mix (**D**) in 2017 on 47, 65, and 99 days after the midpoint of lambing in 2016 and 2017 (L47, L65, and L99).

The percentage of chicory and plantain in the herb-clover mix was greater (*p* < 0.05) in 2016 than in 2017 (Figure 1). Total clover (red clover and white clover) in the herb-clover mix did not differ (*p* > 0.05) between years. Percentage of ryegrass and total clover in the grass-predominant pasture was greater (*p* < 0.05) in 2017 than in 2016. The combined percentage of other grass species was greater (*p* < 0.05) in 2016 than in 2017.

In both years, the herbage mass of herb-clover mix was greater (*p* < 0.05) than that of grass-predominant pasture at the start of the study (L47, Table 1). Thereafter, herbage mass between herb-clover mix and grass-predominant pasture did not differ (*p* > 0.05).

In 2016, at L47, the CP content of herb-clover mix was greater (*p* < 0.05) than grass-predominant pasture. At L65 and L99, however, CP content of herb-clover mix did not differ (*p* > 0.05) from that of grass-predominant pasture (Table 1). At L47, L65 and L99, the NDF content of herb-clover mix was lower (*p* < 0.05) and DMD and ME were greater (*p* < 0.05) than that of grass-predominant pasture. The ADF content of herb-clover mix, however, did not differ (*p* > 0.05) from that of grass-predominant pasture at L47, L65 and L99.

In 2017, the CP content of herb-clover mix was greater (*p* < 0.05) than that of grass-predominant pasture at L47 but did not differ (*p* > 0.05) at L65 and L99. At L47, L65 and L99, the NDF content of herb-clover mix was greater (*p* < 0.05) and DMD was lower (*p* < 0.05) than that of grass-predominant pasture. The ADF content of herb-clover mix did not differ (*p* > 0.05) from that of grass-predominant pasture at L47 and L65 but was greater (*p* < 0.05) at L99. The ME of grass-predominant pasture was greater (*p* < 0.05) than that of herb-clover mix at L47 and L99 but was similar (*p* > 0.05) at L65.

### 3.2. Lamb Live Weight and Liveweight Gain

**Figure 2:**
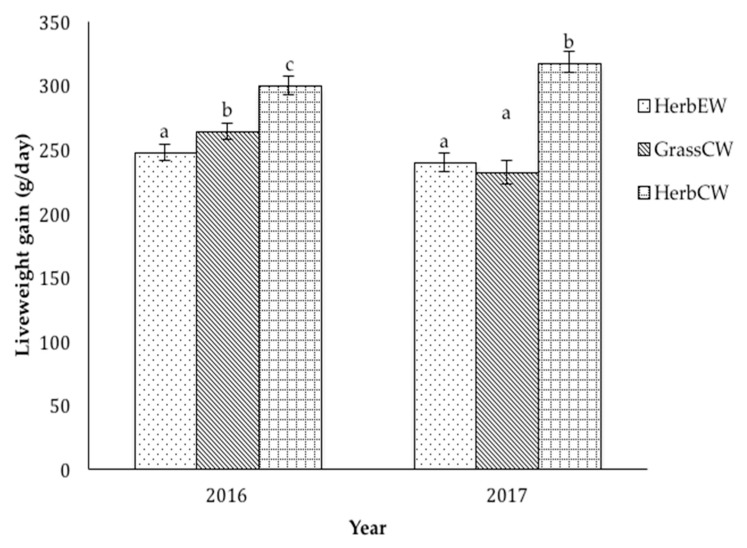
Liveweight gain (g/day) of lambs, from early weaning to conventional weaning, in the Herb_EW_, Herb_CW_ and Grass_CW_ treatments in 2016 and in 2017 (^a–c^ means with different superscripts are significantly different within each year and treatments).

In 2016, the live weights of lambs in the Herb_EW_, Herb_CW_, and Grass_CW_ treatments did not differ (*p* > 0.05) at the start of the study (L53, Table 2). At L65, the live weight of lambs in Herb_CW_ and Grass_CW_ did not differ (*p* > 0.05) but were heavier (*p* < 0.05) than lambs in Herb_EW_. At L99, the live weight of Herb_CW_ lambs was greater (*p* < 0.05) than Grass_CW_ lambs, which in turn was heavier (*p* < 0.05) than Herb_EW_ lambs. Lamb liveweight gains between L53 and L99 in Herb_CW_ treatment were greater (*p* < 0.05) than Grass_CW_, which in turn were greater (*p* < 0.05) than Herb_EW_ (Figure 2).

In 2017, at the start of the study (L51), the live weight of lambs in Herb_EW_, Herb_CW_, and Grass_CW_ did not differ (*p* > 0.05). The live weight of lambs at L65 in Herb_CW_ and Grass_CW_ treatments did not differ (*p* > 0.05) but were heavier (*p* < 0.05) than Herb_EW_ lambs. At L99, the live weight of Herb_CW_ lambs was greater (*p* < 0.05) than both the Herb_EW_ and Grass_CW_ lambs, which did not differ (*p* > 0.05). The liveweight gains of lambs between L51 and L99 in Herb_CW_ treatment were greater (*p* < 0.05) than both Herb_EW_ and Grass_CW_ treatments, which did not differ (*p* > 0.05, Figure 2). Early weaning had no effect on lamb survival as in 2016 one lamb died in each of the Herb_EW_ and Herb_CW_ treatments and no lambs died in 2017.

### 3.3. Ewe Live Weight, Liveweight Gain, and Body Condition Score

In 2016, at the start of the study (L53), the live weights of ewes in each treatment did not differ (*p* > 0.05) (Table 3). At both L65 and L99, the live weights of Herb_EW_ and Herb_CW_ ewes did not differ (*p* > 0.05) but were greater (*p* < 0.05) than Grass_CW_ ewes. The liveweight gain between L53 and L99 of Herb_EW_ ewes (192 ± 15 g/day) and Herb_CW_ ewes (173 ± 15 g/day) did not differ (*p* > 0.05) but was greater (*p* < 0.05) than Grass_CW_ ewes (76 ± 16 g/day).

In 2017, at the start of the study (L51), the live weights of ewes in each treatment did not differ (*p* > 0.05) (Table 3). At L65, ewe live weights did not differ (*p* > 0.05) between all three treatments. At L99, Herb_CW_ ewes were heavier (*p* < 0.05) than both Herb_EW_ and Grass_CW_ ewes, which did not differ (*p* > 0.05). The liveweight gains between L51 and L99 of Herb_EW_ ewes (112 ± 16 g/day) and Herb_CW_ ewes (145 ± 17 g/day) did not differ (*p* > 0.05) but were greater (*p* < 0.05) than Grass_CW_ ewes (78 ± 19 g/day).

In 2016, at L53, the BCS of ewes did not differ (*p* > 0.05) between treatments (Table 4). At L65 and L99, the BCS of Herb_EW_ ewes was greater (*p* < 0.05) than that of Herb_CW_ ewes, which in turn was greater (*p* < 0.05) than Grass_CW_ ewes. In 2017, at L51, BCS of ewes did not differ (*p* > 0.05) between treatments. At L65, the BCS of Herb_CW_ and Grass_CW_ ewes did not differ (*p* > 0.05) but was lower (*p* < 0.05) than Herb_EW_ ewes. At L99, the BCS of Herb_EW_ and Herb_CW_ did not differ (*p* > 0.05) but was greater (*p* < 0.05) than the Grass_CW_ ewes.

## 4. Discussion

The aim of the present study was to determine the impact of weaning lambs early at a minimum live weight of 14 kg, at ~50 days of age, onto a herb-clover mix, on the liveweight of lambs and ewes to a conventional weaning age compared to leaving lambs with their dams unweaned on a herb-clover mix or grass-predominant pasture. Overall, the lambs weaned early onto a herb-clover mix at a minimum live weight of 14 kg gained live weights similar to commercially-reared and conventionally-weaned twin lambs in New Zealand [18]. These results suggest that lambs can be weaned at a minimum of 14 kg live weight, at ~50 days of age, provided a minimum pre-grazing cover of approximately 3000 kg DM/ha of herb-clover mix, without impacting normal growth rates.

The liveweight gain of early-weaned lambs varied between years. In 2016, lambs weaned early onto a herb-clover mix were statistically 800 g lighter at conventional weaning age than lambs that remained with their dams on a grass-predominant pasture. While in 2017, lambs weaned early had similar liveweight gains and weaning weights compared to lambs unweaned on grass-predominant pasture resulting similar live weights at conventional weaning. This suggests that lambs weaned early onto a herb-clover mix have the potential to perform as similar as lambs unweaned on grass-predominant pasture at conventional weaning. The liveweight gain of early-weaned lambs in both years of the present study were similar to those previously reported for lambs weaned at a minimum live weight of 16 kg, at approximately 60 days of age, onto a herb-clover mix [11,19].

The difference in growth rates between early-weaned lambs and unweaned lambs in 2016 and in 2017, however, is of interest as it might indicate when early weaning onto a herb-clover mix is best utilized by farmers. It has been shown that when grass-predominant pasture masses were lower than 1200 kg DM/ha, lamb growth rates were improved by early weaning onto a herb-clover mix [20]. In the present study, pasture mass of grass-predominant pasture was not limiting lamb growth [12], therefore, does not explain the variation of growth rates between early-weaned lambs and unweaned lambs.

In the present study, the nutritional quality of herb-clover mix and grass-predominant pastures differed between years. In 2016, when early-weaned lambs grew slower than unweaned lambs on grass-predominant pasture, ME of herb-clover mix was higher than grass-predominant pasture, while in 2017, when lamb growth did not differ from unweaned lambs on grass-predominant pasture, ME of herb-clover mix was lower than grass-predominant pasture. Combined this suggests that the variation of ME is not the reason for the observed differences of lambs liveweight gains between years. In both years of the study, the CP of herb-clover mix was greater than grass-predominant pasture at the start of the experiment but did not differ throughout the remainder of the study period. Although the CP of both herbages were at the lower end of that is required for lamb growth (15%–18% CP) in both years [21]. The variation in the CP content of the herb-clover mix and grass-predominant pasture does not also explain the variable liveweight gains.

The botanical composition of herbage changed between years within the herbage types, resulting in variation in the availability of the different plant species in both the herb-clover mix and grass-predominant pasture. Lambs have been shown to preferentially select certain plant species when offered as cut-and-carried fresh forage [9] and under grazing [22], therefore, the variation of the botanical composition of herbage between years could have affected the overall lamb nutrient intake, thus their growth rates and their live weights at conventional weaning. The botanical composition of the herbages was determined using a quadrat-cut method and therefore, may not have reflected what accurately what the lambs actually consumed. In the future, to understand the potential causes of variation, studies should attempt to determine which plants the animals are choosing and then undertake herbage quality analysis based on this. Although the data available in the present study makes it is difficult to explain the variation of live weights at conventional weaning age over two years, the difference of lamb weights was only 800 g which is unlikely to have a significant impact on the overall production system. Further research, however, is required to identify conditions that allow early weaned lambs to grow consistently as fast as those unweaned lambs offered grass-predominant pasture.

In both years of the current study, unweaned lambs on herb-clover mix grew faster, and were heavier at conventional weaning, than lambs unweaned on grass-predominant pasture. This has also been reported in previous studies [3,11]. In addition, a greater milk production of twin and triplet-rearing ewes has been reported when grazing the herb-clover mix compared with grass-predominant pasture [1]. Therefore, the greater liveweight gain of unweaned lambs on herb-clover mix than on grass-predominant pasture in both years could have been due to higher milk production of ewes on herb-clover mix, resulting in greater lamb milk intake as well as increased herbage intake of lambs, through preferential selection of plant species [10], or a combination of all these variables. Unfortunately, in the current study, neither milk nor herbage intake of lambs was measured.

In both years of the current study, unweaned lambs on herb-clover mix had greater liveweight gains than early-weaned lambs. This was also found in previous studies [3,11]. Early-weaned lambs had access to only herbage post-weaning while unweaned lambs had access to both milk and herbage. This suggests that if there is enough herbage, lambs should be left with their dams on herb-clover mix to achieve the greatest growth rates. Early weaning onto an herb-clover mix, however, can allow farmers to graze lambs at much greater stocking density on herb-clover mix, if only a small area is available, which is a common scenario in New Zealand, and thus to improve pasture utilization efficiency.

Early weaned ewes had greater liveweight gains and BCS than unweaned ewes on grass-predominant pasture in both years. Early-weaned ewes were managed along with unweaned ewes and lambs on the grass-predominant pasture until conventional weaning age, thus suggesting that the greater live weight and BCS observed was due to the cessation of lactation, reducing the energy requirement of the ewe [23]. Both weaned and unweaned ewes on herb-clover mix had similar liveweight gains to conventional weaning in both years of the study. Early weaning, therefore, can be used as a technique to improve ewe live weights and potentially their future performance as live weight at breeding has a positive impact on ewe reproductive performance [24].

## 5. Conclusions

Lambs weaned early at a minimum live weight of 14 kg, at ~50 days of age, onto a herb-clover mix have the potential to achieve similar liveweight gains as lambs unweaned on grass-predominant pasture. Early weaning can allow ewes to gain greater live weights and BCS compared to conventional weaning on grass-predominant pasture. However, further research is required to determine how a consistent lamb growth response can be achieved by early weaning onto an herb-clover mix.

## Figures and Tables

**Table 1:** Herbage mass (HM), crude protein (CP), neutral detergent fiber (NDF), acid detergent fiber (ADF), dry matter digestibility (DMD), and metabolizable energy content (ME) of herbage samples collected 47, 65 and 99 days after the midpoint of lambing in 2016 and 2017 (L47, L65 and L99) (least-squares mean ± SEM).

Herbage Type		HM (kg DM/ha)	CP (%)	NDF (%)	ADF (%)	DMD (%)	ME (MJ/Kg)
2016							
Herb-clover mix	L47	3782 ^b^ ± 481	21.2 ^c^ ± 1.0	32.1 ^a^ ± 2.0	26.9 ^a^ ± 2.3	72.9 ^e^ ± 0.8	10.6 ^d^ ± 0.1
	L65	3362 ^ab^ ± 481	15.3 ^a^ ± 1.0	40.8 ^b^ ± 2.0	33.3 ^ab^ ± 2.3	70.3 ^d^ ± 0.8	10.2 ^c^ ± 0.1
	L99	3390 ^ab^ ± 481	13.4 ^a^ ± 1.0	45.6 ^c^ ± 2.0	36.2 ^b^ ± 2.3	68.5 ^d^ ± 0.8	9.8 ^c^ ± 0.1
Grass-predominant pasture	L47	2362 ^a^ ± 481	19.5 ^b^ ± 1.0	51.0 ^d^ ± 2.0	29.9 ^a^ ± 2.3	65.0 ^c^ ± 0.8	9.2 ^b^ ± 0.1
	L65	3308 ^ab^ ± 481	14.3 ^a^ ± 1.0	60.7 ^e^ ± 2.0	39.0 ^b^ ± 2.3	62.4 ^b^ ± 0.8	8.6 ^a^ ± 0.1
	L99	3270 ^ab^ ± 481	13.5 ^a^ ± 1.0	62.0 ^e^ ± 2.0	38.3 ^b^ ± 2.3	60.7 ^a^ ± 0.8	8.7 ^a^ ± 0.1
2017							
Herb-clover mix	L47	3221 ^b^ ± 481	17.3 ^c^ ± 1.0	46.7 ^c^ ± 2.0	26.5 ^a^ ± 2.3	65.7 ^b^ ± 0.8	9.5 ^ab^ ± 0.1
	L65	2994 ^ab^ ± 481	9.8 ^a^ ± 1.0	48.4 ^c^ ± 2.0	25.5 ^a^ ± 2.3	65.4 ^ab^ ± 0.8	9.8 ^bc^ ± 0.1
	L99	3048 ^b^ ± 481	15.5 ^bc^ ± 1.0	53.7 ^d^ ± 2.0	30.5 ^b^ ± 2.3	63.4 ^a^ ± 0.8	9.1 ^a^ ± 0.1
Grass-predominant pasture	L47	1899 ^a^ ± 481	12.0 ^a^ ± 1.0	32.7 ^a^ ± 2.0	26.2 ^a^ ± 2.3	72.0 ^d^ ± 0.8	10.6 ^d^ ± 0.1
	L65	2659 ^ab^ ± 481	10.4 ^a^ ± 1.0	39.7 ^b^ ± 2.0	29.6 ^a^ ± 2.3	68.9 ^c^ ± 0.8	10.1 ^c^ ± 0.1
	L99	2680 ^ab^ ± 481	13.0 ^b^ ± 1.0	31.4 ^a^ ± 2.0	23.7 ^a^ ± 2.3	73.1 ^d^ ± 0.8	10.6 ^d^ ± 0.1

^a–e^ Means with different superscripts within columns are significantly different across years and treatments (*p* < 0.05).

**Table 2:** Live weight of lambs in Herb_EW_, Herb_CW_ and Grass_CW_ treatments at L53, L65 and L99 in 2016 and at L51, L65 and L99 in 2017 (least-squares mean ± SEM).

Herbage Treatment	Lamb Live Weight (kg)					
	n		n		n	
	2016					
		L53		L65		L99
Herb_EW_	54	19.3 ± 0.1 ^a^	53	21.5 ± 0.2 ^b^	53	30.7 ± 0.3 ^d^
Herb_CW_	45	19.5 ± 0.1 ^a^	46	23.6 ± 0.2 ^c^	45	33.2 ± 0.4 ^f^
Grass_CW_	48	19.4 ± 0.1 ^a^	49	23.3 ± 0.2 ^c^	50	31.5 ± 0.3 ^e^
	2017					
		L51		L65		L99
Herb_EW_	46	19.0 ± 0.2 ^a^	45	21.5 ± 0.2 ^b^	46	30.5 ± 0.4 ^d^
Herb_CW_	42	19.3 ± 0.2 ^a^	41	23.0 ± 0.2 ^c^	42	34.5 ± 0.4 ^e^
Grass_CW_	34	19.1 ± 0.2 ^a^	33	22.7 ± 0.2 ^c^	34	30.2 ± 0.4 ^d^

L, days after the midpoint of lambing; Herb_EW_, Early weaning onto unrestricted allowance of herb-clover mix; Herb_CW_, Lambs and dams offered an unrestricted allowance of herb-clover mix until conventional weaning age; Grass_CW_, Lambs and dams offered unrestricted allowance of grass-predominant pasture until conventional weaning age. ^a–f^ Means with different superscripts are significantly different within each year and treatments. In each year, lambs in HerbEW grazed with lambs and their ewes in HerbCW. Lambs in GrassCW grazed with both GrassCW and HerbEW ewes.

**Table 3:** Live weight of ewes in Herb_EW_, Herb_CW_ and Grass_CW_ treatments at L53, L65 and L99 in 2016 and at L51, L65 and L99 in 2017 (least-squares mean ± SEM).

Herbage Treatment	Ewe Live Weight (kg)					
	n		n		n	
	2016					
		L53		L65		L99
Herb_EW_	27	71.3 ± 1.3 ^a^	25	77.1 ± 1.3 ^b^	25	80.3 ± 1.3 ^c^
Herb_CW_	23	72.2 ± 1.4 ^ab^	23	76.5 ± 1.4 ^b^	23	80.0 ± 1.4 ^c^
Grass_CW_	25	71.2 ± 1.3 ^a^	24	73.6 ± 1.3 ^a^	23	75.1 ± 1.4 ^b^
	2017					
		L51		L65		L99
Herb_EW_	23	67.9 ± 1.4 ^a^	23	69.2 ± 1.4 ^b^	22	73.3 ± 1.4 ^b^
Herb_CW_	21	69.4 ± 1.5 ^ab^	20	70.5 ± 1.5 ^b^	21	76.4 ± 1.5 ^c^
Grass_CW_	17	67.7 ± 1.6 ^a^	16	69.2 ± 1.4 ^ab^	17	72.4 ± 1.6 ^b^

L, days after the midpoint of lambing; Herb_EW_, Early weaning onto unrestricted allowance of herb-clover mix; Herb_CW_, lambs and dams offered an unrestricted allowance of herb-clover mix until conventional weaning age; Grass_CW_, lambs and dams offered unrestricted allowance of grass-predominant pasture until conventional weaning age. ^a–c^ Means with different superscripts are significantly different within each year and treatments. In each year, ewes in Herb_EW_ grazed with lambs and their ewes in Grass_CW_. Ewes in Herb_CW_ grazed with their lambs and the weaned lambs in the Herb_EW_ treatment.

**Table 4:** Body condition score of ewes in the Herb_EW_, Herb_CW_ and Grass_CW_ treatments at L53, L65, and L99 in 2016 and at L51, L65, and L99 in 2017 (Results displayed as back transformed logit mean and 95% confidence interval).

Herbage Treatment	Ewe Body Condition Score					
	n		n		n	
	2016					
		L53		L65		L99
Herb_EW_	26	2.3 (2.2–2.4) ^a^	25	3.1 (2.9–3.3) ^d^	25	3.4 (3.2–3.6) ^e^
Herb_CW_	23	2.1 (2.0–2.2) ^a^	23	2.8 (2.6–2.9) ^c^	23	3.0 (2.8–3.2) ^d^
Grass_CW_	25	2.1 (2.0–2.3) ^a^	24	2.5 (2.4–2.6) ^b^	23	2.5 (2.4–2.7) ^b^
	2017					
		L51		L65		L99
Herb_EW_	23	2.9 (2.8–3.1) ^a^	23	3.3 (3.1–3.6) ^b^	22	3.4 (3.2–3.5) ^b^
Herb_CW_	21	2.7 (2.5–2.9) ^a^	20	2.8 (2.6–3.0) ^a^	21	3.3 (3.1–3.4) ^b^
Grass_CW_	17	2.8 (2.6–3.0) ^a^	16	2.6 (2.2–3.0) ^a^	17	2.9 (2.7–3.1) ^a^

L, days after the midpoint of lambing; Herb_EW_, Early weaning onto unrestricted allowance of herb-clover mix; Herb_CW_, lambs and dams offered an unrestricted allowance of herb-clover mix until conventional weaning age; Grass_CW_, lambs and dams offered unrestricted allowance of grass-predominant pasture until conventional weaning age. ^a–d^ Means with different superscripts are significantly different within each year and treatments. In each year, ewes in Herb_EW_ grazed with lambs and their ewes in Grass_CW_. Ewes in Herb_CW_ grazed with their lambs and the weaned lambs in the Herb_EW_ treatment.
